# Down‐regulation of long non‐coding RNA MEG3 promotes Schwann cell proliferation and migration and repairs sciatic nerve injury in rats

**DOI:** 10.1111/jcmm.15368

**Published:** 2020-05-20

**Authors:** Yongbin Ma, Dongwang Zhai, Wenzhe Zhang, Huanyan Zhang, Liyang Dong, Yuepeng Zhou, Dingqi Feng, Yu Zheng, Ting Wang, Chaoming Mao, Xuefeng Wang

**Affiliations:** ^1^ Department of Central Laboratory The Affiliated Hospital of Jiangsu University Zhenjiang China; ^2^ Department of Pathogen Biology & Immunology Jiangsu Key Laboratory of Pathogen Biology Nanjing Medical University Nanjing China; ^3^ Department of Neurology Laboratory Jintan Hospital Jiangsu University Jintan China; ^4^ Department of Clinical Laboratory Aerospace Center Hospital Peking University Beijing China; ^5^ Department of Nuclear Medicine and Institute of Oncology The Affiliated Hospital of Jiangsu University Zhenjiang China

**Keywords:** long non‐coding RNA MEG3, migration, proliferation, Schwann cells, sciatic nerve transection

## Abstract

Peripheral nerve injury and regeneration are complex processes and involve multiple molecular and signalling components. However, the involvement of long non‐coding RNA (lncRNA) in this process is not fully clarified. In this study, we evaluated the expression of the lncRNA maternally expressed gene 3 (MEG3) in rats after sciatic nerve transection and explored its potential mechanisms. The expression of lncRNA MEG3 was up‐regulated following sciatic nerve injury and observed in Schwann cells (SCs). The down‐regulation of lncRNA MEG3 in SCs enhanced the proliferation and migration of SCs via the PTEN/PI3K/AKT pathway. The silencing of lncRNA MEG3 promoted the migration of SCs and axon outgrowth in rats after sciatic nerve transection and facilitated rat nerve regeneration and functional recovery. Our findings indicated that lncRNA MEG3 may be involved in nerve injury and injured nerve regeneration in rats with sciatic nerve defects by regulating the proliferation and migration of SCs. This gene may provide a potential therapeutic target for improving peripheral nerve injury.

## 1 INTRODUCTION

Peripheral nerve injury is a major clinical problem in adults due to its high disability and mortality.^1^ Although the peripheral nerve system (PNS) has axonal regeneration ability, PNS often shows an incomplete functional recovery following nerve injury.^2^ After this type of injury, the lesion site undergoes a series of pathophysiological alterations, including axonal degeneration and proliferation and migration of Schwann cells (SCs) to form Büngner bands, which provide a suitable microenvironment and facilitate axonal regeneration.^3^, ^4^ However, the molecular mechanism of nerve injury and regeneration in PNS has not been fully clarified.

Non‐coding RNAs (ncRNAs) especially long ncRNAs (lncRNAs) are differentially expressed in the central and peripheral nerve injury sites and possess regulatory roles in neural injury and regeneration.^5^ Zhao and colleagues reported that 322 lncRNAs are differentially expressed in hypoxic‐ischemic brains, and the BC088414 lncRNA is substantially up‐regulated following brain damage, suggesting its involvement in the pathogenesis of central nervous system injury.^6^ Similarly, 105 differentially expressed lncRNAs and down‐regulated BC089918 and uc.217 are observed in the rat dorsal root ganglion after sciatic nerve injury.^7^ The silencing of BC089918 and uc.217 can promote the neurite outgrowth of neurons in the dorsal root ganglion, revealing that lncRNAs play an important role in peripheral nerve injury.^7^, ^8^ In addition, NONMMUG014387 lncRNA promotes the proliferation of SCs following peripheral nerve injury.^9^ However, the role of lncRNAs in peripheral nerve injury remains unclear, and other key lncRNAs must be explored.

The maternally expressed gene 3 (MEG3) is located in the human 14q32 chromosome, named as gene trap locus 2 in mouse, and widely investigated in tumour cases.^10^, ^11^ Accumulating evidence suggests that MEG3 is a tumour‐suppressing lncRNA and down‐regulated in multiple malignancies.^12^ However, its role in peripheral nerve injury and how it modulates injured nerve regeneration remain to be clarified.

In this study, MEG3 is expressed in the SCs of rats and up‐regulated after sciatic nerve transection. Silencing MEG3 in SCs can enhance the proliferation and migration of these cells via the phosphatase and tensin homolog (PTEN)/phosphoinositide 3‐kinase (PI3K)/AKT pathway and promote rat nerve regeneration and functional recovery. Thus, MEG3 may be involved in nerve injury and injured nerve regeneration in rats with sciatic nerve defects and may provide a potential therapeutic target for peripheral nerve injury.

## 2 MATERIALS AND METHODS

### 2.1 Rat surgery

A total of 24 adult male Sprague‐Dawley (SD) rats (220‐230 g) were anaesthetized with xylazine and ketamine as previously described.^13^ The sciatic nerve at 1 cm above the bifurcation into the tibial and common fibular nerves was exposed and cut using ophthalmic scissors, and the incision was closed with 4‐0 nylon sutures. The proximal stumps of the sciatic nerve from each group of animals were collected at different time points (0, 1, 4 and 7 days) after the injury. All experimental procedures involving animals were approved by the Institutional Animal Care and Use Committee of Jiangsu University (Permit Number: JSU 17‐112).

### 2.2 Quantitative real‐time polymerase chain reaction (qRT‐PCR)

Total RNA was isolated from the sciatic nerve segment by using the Trizol reagent (Invitrogen) and reverse‐transcribed into cDNA by using the Prime‐Script RT reagent Kit (Genecopoeia, Germantown, MD, USA). PCR was performed using the All‐in‐one™qPCR Mix (Genecopoeia) and the primers purchased from Genecopoeia on the ABI system (Applied Biosystems, Foster City, CA, USA) following manufacturer's protocol. The relative expression of lncRNA MEG3 was calculated using the comparative 2^−ΔΔCt^ method.

### 2.3 Fluorescent in situ hybridization (FISH)

Cy3‐labelled rat lncRNA MEG3 probes (Probe sequence: 5ʹ‐CY3‐CACAGGAAGACGCGACGGGCCAGGGTA‐CY3‐3ʹ) were synthesized by Servicebio. The nerve tissue sections were treated with 20 μg/mL proteinase K for 30 minutes at 37°C, and hybridization and washing were conducted as previously described.^14^ The MEG3 expression in the rat sciatic nerve was performed using FISH as described in a previous study.^15^ Briefly, Cy3‐labelled MEG3 was used to detect the injured (model group) and the normal (normal group) rat sciatic nerve tissues. The negative probe was utilized as the negative control (Negative group). The images were obtained using the Nikon ECLIPSE CI microscope (ECLIPSE CI, Nikon, Japan). Cy3‐labelled lncRNA MEG3 displayed red fluorescence, which represented the expression level of lncRNA MEG3. A high intensity indicated high MEG3 expression.

### 2.4 Isolation and characterization of SCs

Schwann cells were isolated and cultured following a previously published protocol with slight modifications.^16^ The bilateral sciatic nerves and brachial plexus of 2‐ to 3‐day‐old SD rats were collected, minced, and digested with trypsin and collagenase type I.^16^ After digestion, the cells were cultured using the SC culture medium (ScienCell, USA) and purified with 5 μg/mL cytosine arabinoside (Sigma, USA).^16^

### 2.5 Immunofluorescence

The SCs were fixed in 4% paraformaldehyde for 30 minutes at 4°C, blocked and incubated with monoclonal anti‐S100 (1:200),anti‐GFAP (1:200) and anti‐Ki67 (1:200, Servicebio, Wuhan, China) primary antibodies overnight at 4°C. The Alexa Fluor 488 goat anti‐rabbit (1:200, Servicebio) or Cy3 goat anti‐rabbit (1:200, Servicebio) was used as the secondary antibody, and DAPI (Servicebio) was utilized to detect the nucleus. The images were obtained using an inverted fluorescence microscope (CKX‐53, Olympus, Japan).

### 2.6 Cell transfection

The SCs were transfected with lncRNA MEG3 siRNA with Lipofectamine RNAi MAX transfection reagent (Invitrogen, Carlsbad CA, USA) in Opti‐MEM (Invitrogen) in accordance with the manufacturer's protocol. The synthetic MEG3‐siRNA and the control were purchased from Genecopoeia.

### 2.7 Cell counting kit‐8 (CCK‐8) proliferation assay

The cell proliferation assay was performed using the CCK‐8 solution (Beyotime, Nantong, China) following the manufacturer's instructions. The SCs transfected with MEG3‐siRNA or control were seeded on a 96‐well plate with a SC medium. After being cultured for 48 hours, the cells were treated with CCK‐8 solution for 2 hours, and the optimal density (OD) at 450 nm was measured using a microplate reader (Synergy HT, BioTek, Biotek Winooski, VT).

### 2.8 Transwell migration assay

The migration capabilities of SCs were examined using the Transwell migration assay. The SCs transfected with MEG3‐siRNA or control in serum‐free medium (100 μL, 1 × 10^5^ cells) were seeded on the upper chamber of the Transwell, and the lower chamber of the Transwell was added with complete medium (600 μL). After being cultured for 12 hours, the cells that migrated from the top chamber to the bottom surface were stained with 0.1% crystal violet. The images were obtained and counted using the ICC50 HD microscope (Leica Microsytems) and the Image J software.

### 2.9 Western blot analysis

Proteins were extracted from proximal nerve tissue (5 mm long) or SCs transfected with MEG3‐siRNA or control for Western blot analysis as previously described.^13^ The primary antibodies were as follows: anti‐PTEN (Cell Signaling Technology, Danvers, MA, USA) (1:1000), anti‐PI3K (CST, 1:1000), anti‐p‐PI3K (Affinity Biosciences, OH, USA) (1:1000), anti‐AKT (CST, 1:1000), anti‐p‐AKT (CST, 1:1000), anti‐S6 (CST, 1:1000), anti‐p‐S6 (CST, 1:1000) and β‐actin (CST, 1:1000). Anti‐rabbit IgG (CST, 1:2000) was used as the second antibody, and β‐actin was used as the internal control.

### 2.10 SC migration and axon outgrowth in vivo

The SD rat sciatic nerve injury model was constructed as previously described.^13^ The sciatic nerves were exposed and removed (3 mm) to form a 5 mm‐long sciatic nerve gap between the distal and proximal nerve stumps. A silicone rubber tube was inserted into the gap. 0.2 OD (15 μL) MEG3‐siRNA or control (Ribobio, Guangzhou, China) mixed with Matrigel (1:1 v/v) (BD Biosciences, Billerica, MA) was slowly injected into the conduit lumen by using a pre‐cooled microsyringe in accordance with the previous description.^14^, ^17^ The incision was then closed in a routine fashion. The rats were killed 15 days after the surgery, and the new nerves in the conduit lumen were harvested, embedded in paraffin and sectioned. The migration of SCs and axon outgrowth were assessed using immunofluorescence staining with anti‐SOX10 (red) (Abcam, 1:500) and anti‐β‐Tubulin III (green) (1:100, Servicebio) antibodies.

### 2.11 Walking track analysis

The model of sciatic nerve transection in rat was constructed as previously described.^13^ Walking track analysis was performed, and the sciatic function index (SFI) was measured at 7, 14, 21 and 28 days post‐neurorrhaphy based on the basis of a previously described protocol.

### 2.12 Statistical analysis

All data were presented as mean ± standard error of the mean (SEM). Statistical analyses were performed using Student's *t* test by using the GraphPad Prism V 5.0 (GraphPad, San Diego, CA, USA). *P < *0.05 was regarded as statistically significant.

## 3 RESULTS

### 3.1 lncRNA MEG3 expression is up‐regulated after sciatic nerve injury

**FIGURE 1:**
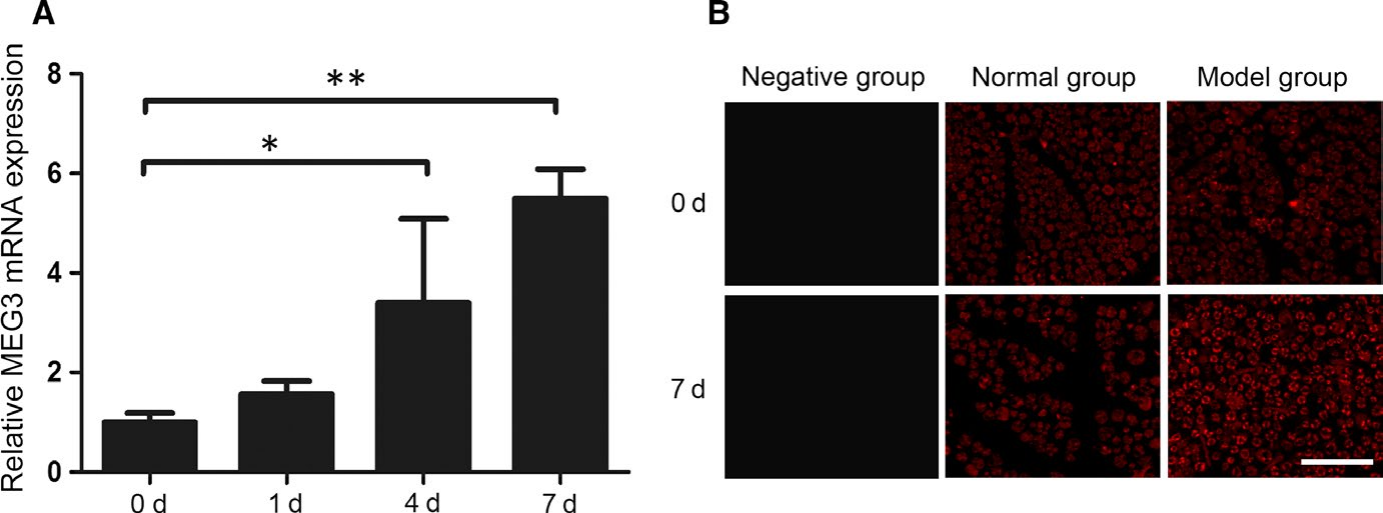
Expression and localization of MEG3 in nerve tissues. A, Detection of MEG3 expression after sciatic nerve injury at 0, 1, 4 and 7 d by qRT‐PCR, n = 6. B, Detection of MEG3 expression in sciatic nerve sections at 0 and 7 d after nerve injury by FISH. Negative group: negative probe; Normal group: normal rat sciatic nerve; Model group: injured rat sciatic nerve, n = 3. Scale bar = 50 μm. Values are presented as mean ± SEM. *P < 0.05, **P < 0.01

Rat sciatic nerve segments were collected for MEG3 analysis by qRT‐PCR to evaluate the MEG3 expression after sciatic nerve injury. As shown in Figure 1A, the MEG3 expression has remarkably increased at days 4 and 7 after sciatic nerve injury compared with that at day 0 (control) and reached its peak on the seventh day (Figure 1A). In situ hybridization has further revealed that MEG3 expression in SCs and that MEG3 is significantly up‐regulated on the seventh day after sciatic nerve injury (Figure 1B).

### 3.2 Localization of lncRNA MEG3 in SCs

**FIGURE 2:**
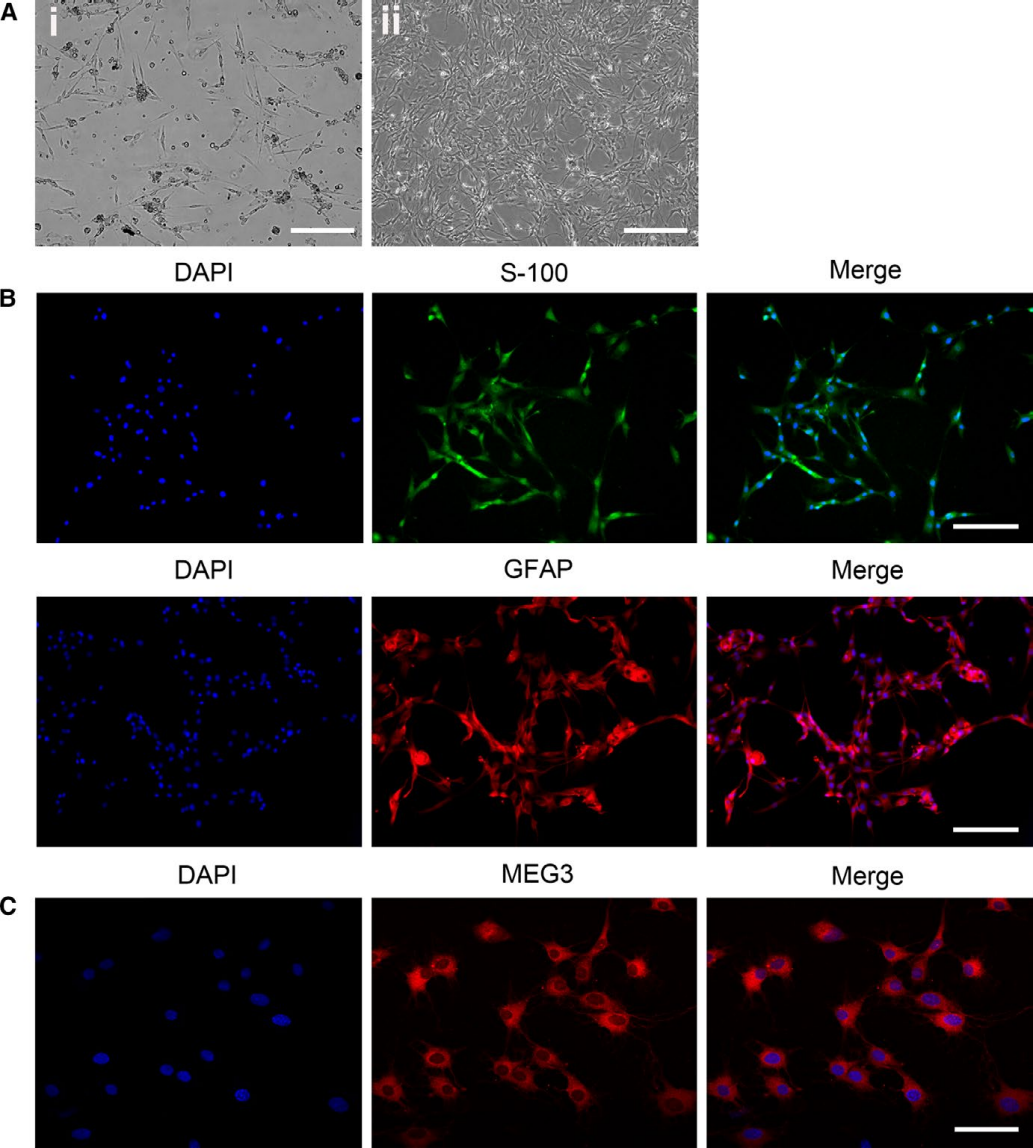
Localization of MEG3 in SCs. A, Morphology of SCs (passages 0 and 5) observed using a light microscope (Ai: Scale bar = 100 μm; Aii: Scale bar = 200μm). B, Images of S‐100 (green) and GFAP (red) antibody staining for SC identification. Nuclei were stained with DAPI (blue), Scale bar = 100 μm. C, Localization analysis of MEG3 by FISH experiment. Nuclei were stained with DAPI (blue). Scale bar = 50 μm

Primary SCs were isolated and cultured to detect and verify the MEG3 expression in SCs through the FISH experiment. As shown in Figure 2A, adherent cells display long spindle‐like shapes after 1 day of initial culture (Figure 2Ai) and show uniform morphology after the purification of the fourth generation (Figure 2Aii). S‐100 and GFAP are considered as identification markers of SCs. As shown in Figure 2B, almost all cells are S‐100‐ and GFAP‐positive, indicating that the extracted cells are SCs. The FISH experiment reveals that MEG3 is localized in the SC cytoplasm (Figure 2C).

### 3.3 lncRNA MEG3 knockdown promotes SC proliferation and migration by regulating the PTEN/PI3K/AKT pathway

**FIGURE 3:**
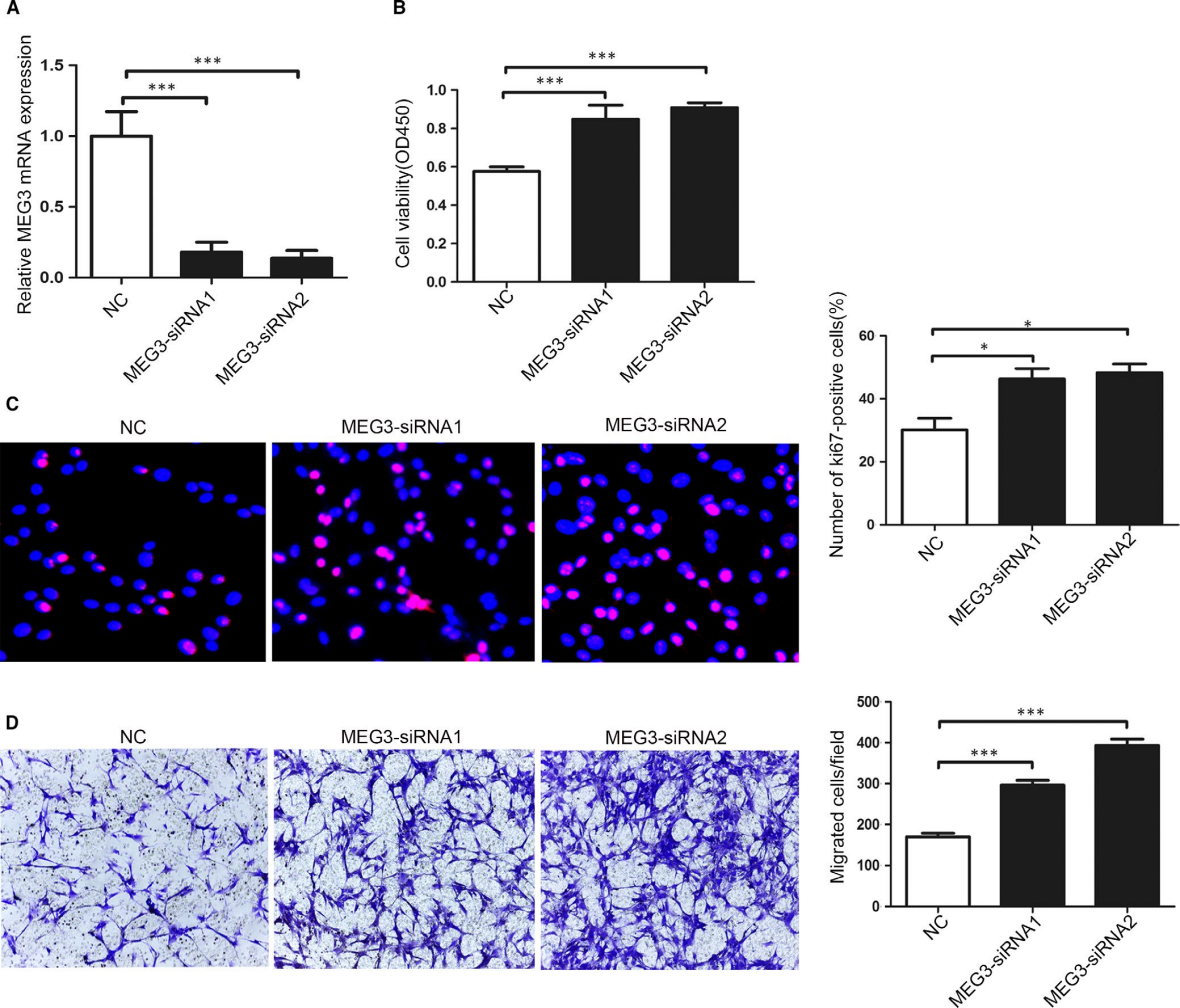
MEG3 affects the proliferation and migration of SCs. A, Specific MEG3‐siRNAs decrease MEG3 expression in SCs as detected by qRT‐PCR. B, CCK8 analysis of SC proliferation after transfection with MEG3‐siRNAs. C, Representative images (left) and quantification data (right) of Ki67 staining (red). Nuclei were stained with DAPI (blue), n = 5. D, Representative images (left) and quantification data (right) of Transwell migration assay, n = 5. Values are presented as mean ± SEM. *P < 0.05, ***P < 0.001

The silencing of MEG3 was performed by transfecting specific siRNAs into SCs to further investigate the role of MEG3 in SCs. Figure 3A illustrates that siRNA1 and siRNA2 have significantly decreased the mRNA expression of MEG3 compared with the NC control. After transfection for 48 hours, the cell proliferation assay shows that the group subjected to MEG3 knockdown can noticeably promote SC proliferation compared with that in the control group (Figure 3B). Ki67 staining has further revealed that MEG3 silencing promotes SC proliferation (Figure 3C). Furthermore, MEG3 down‐regulation can promote the migration of SCs, as indicated by the Transwell migration assay (Figure 3D).

**FIGURE 4:**
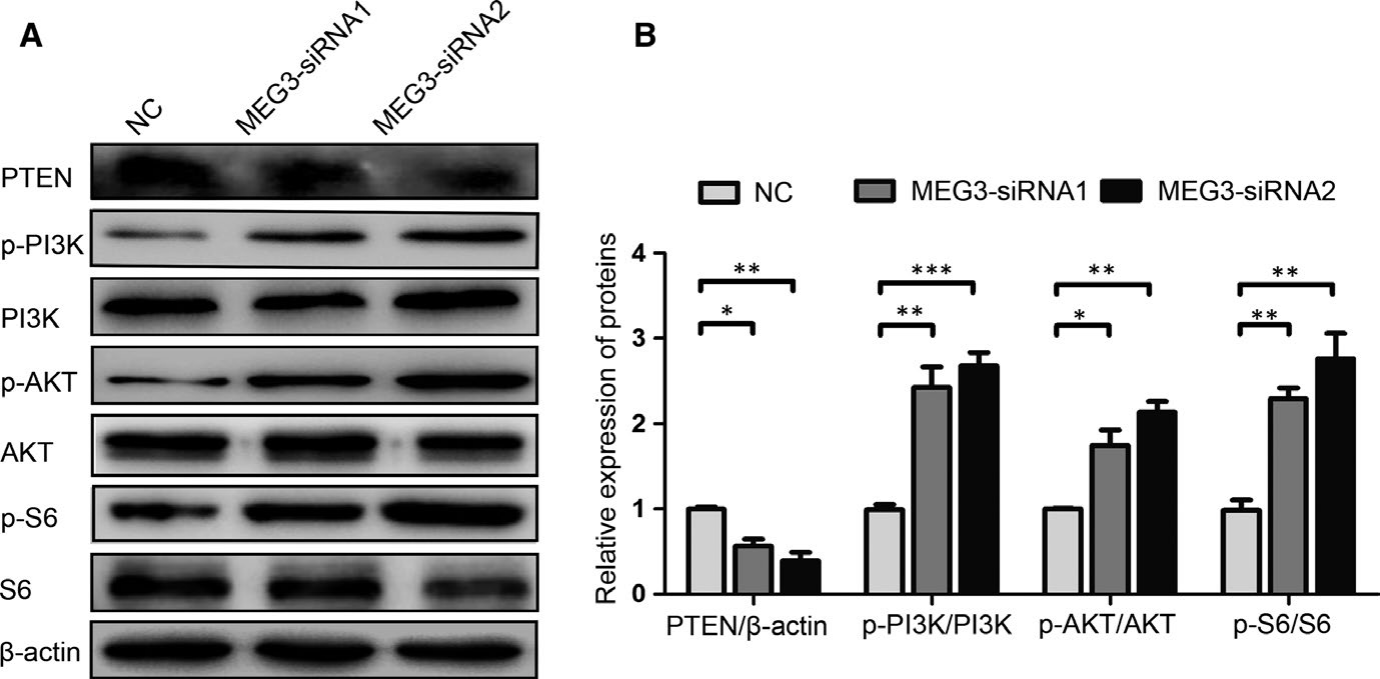
MEG3 regulates the PTEN/PI3K/AKT signalling in primary SCs. Western blot analysis of PTEN, p‐PI3K, PI3K, p‐AKT, AKT, p‐S6 and S6 expression levels after MEG3 siRNA transfection in SCs. A, Representative blot. B, Bar graph of the relative expression level of proteins. Values are obtained from three independent experiments and presented as mean ± SEM. *P < 0.05, **P < 0.01, ***P < 0.001

The PTEN/PI3K/AKT signalling regulates cell proliferation and migration.^18^, ^19^ Thus, the PTEN/PI3K/AKT pathway was used to investigate whether the promotion of SC proliferation and migration was mediated by MEG3 knockdown. The expression of signal molecules in SCs after being transfected with MEG3‐siRNA or control for 48 hours was examined by Western blot analysis. As shown in Figure 4, the MEG3 siRNA transfection down‐regulates the expression of PTEN but up‐regulates those of p‐PI3K, p‐AKT and p‐S6 relative to those in the NC control (Figure 4A,B). These results suggest that MEG3 knockdown promotes SC proliferation and migration by regulating the PTEN/PI3K/AKT pathway.

### 3.4 lncRNA MEG3 knockdown promotes SC proliferation and migration and axon outgrowth in vivo

**FIGURE 5:**
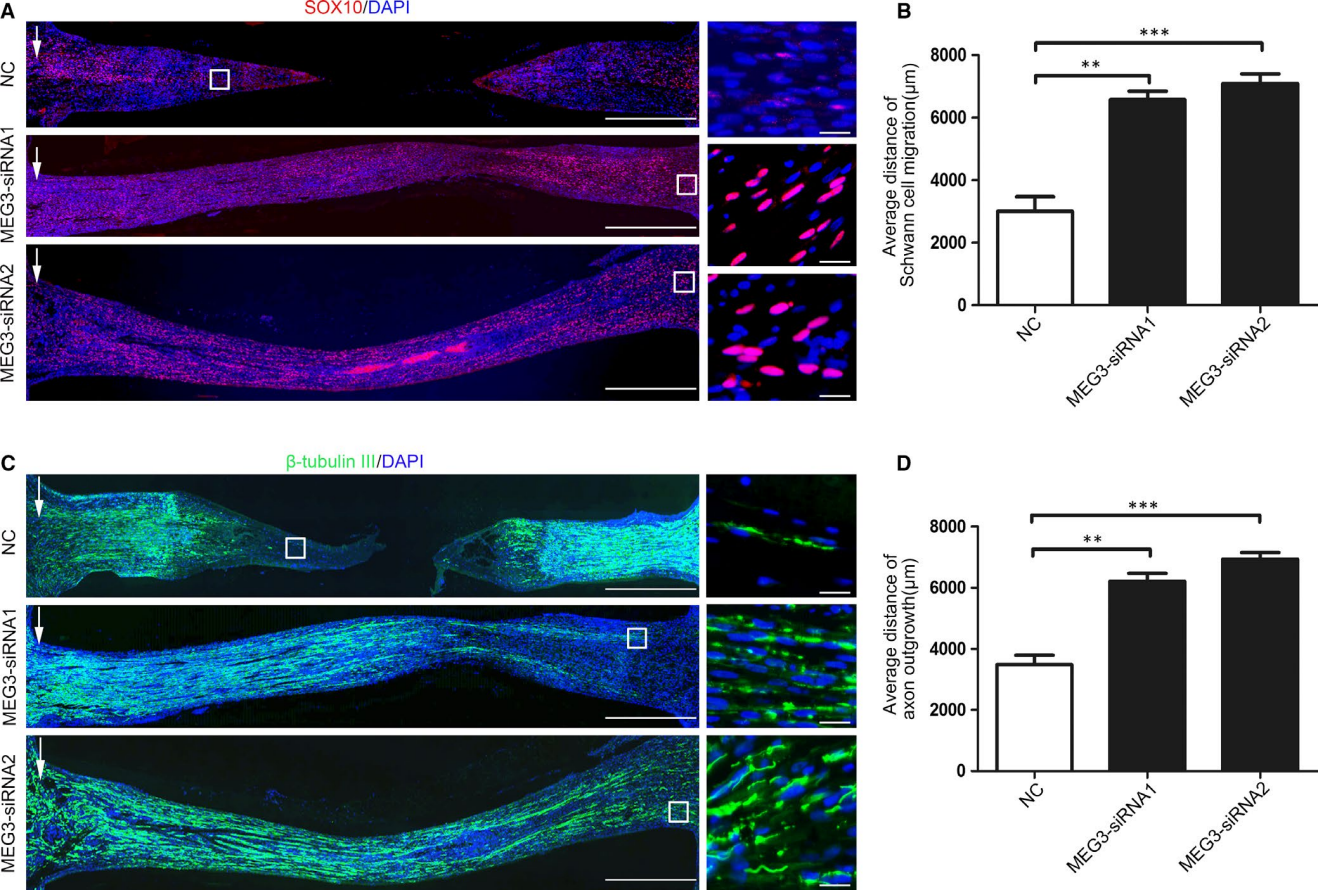
MEG3 affects Schwann cell migration and axon outgrowth in rats after sciatic nerve transection. A, Immunofluorescence detection of the migration distance of Schwann cells in the MEG3‐siRNA and control groups. Red and blue colours represent SOX10‐positive Schwann cells and cell nuclei, respectively. The white arrow represents the edge of the proximal stump and the distance of Schwann cell migration from the edge of the proximal stump (white box), bar = 1000 μm. The image on the right represents the high‐magnification image in the white box on the left, bar = 50 μm. B, Quantitative results showing the average migration distance of Schwann cells, n = 3. C, Images of β‐Tubulin III antibody staining (green) for the detection of regenerated axons. Nuclei were stained with DAPI (blue). The white arrow represents the edge of the proximal stump and the end of axon outgrowth (white box), bar = 1000 μm. The image on the right represents the high‐magnification image in the white box on the left, bar = 50 μm. D, Quantitative results showing average distance of axon outgrowth in silicone conduits, n = 3. Values are presented as mean ± SEM. **P < 0.01, ***P < 0.001

A rat sciatic nerve injury model was established to determine whether MEG3 can also regulate SC migration in vivo, and a silicone‐based nerve guidance conduit was administered to the specific MEG3‐siRNA and control groups. The SCs were detected by immunostaining for the specific marker SOX10 in the longitudinal sections of the regenerated sciatic nerves 18 days after surgery. As shown in Figure 5A and 5B, the MEG3 siRNA injection significantly promotes the proliferation and migration of SCs because of the increased distance of the migrated SCs in the MEG3 siRNA injection group. By contrast, the SCs from the control group have a short migration distance, and the proximal and distal nerves are not connected in sections. Moreover, MEG3 siRNA injection significantly promotes axonal regeneration because the β‐tubulin III expression in the group injected with MEG3 siRNA is significantly up‐regulated compared with that in the NC control group. This finding suggests that MEG3 siRNA injection increases the distance of axon outgrowth in rats with sciatic nerve injury (Figure 5C,D). These results suggest that MEG3 knockdown can promote nerve injury regeneration by regulating the proliferation and migration of SCs in vivo.

### 3.5 lncRNA MEG3 knockdown promotes the functional recovery of the sciatic nerve through regulating the PTEN/PI3K/AKT signalling

**FIGURE 6:**
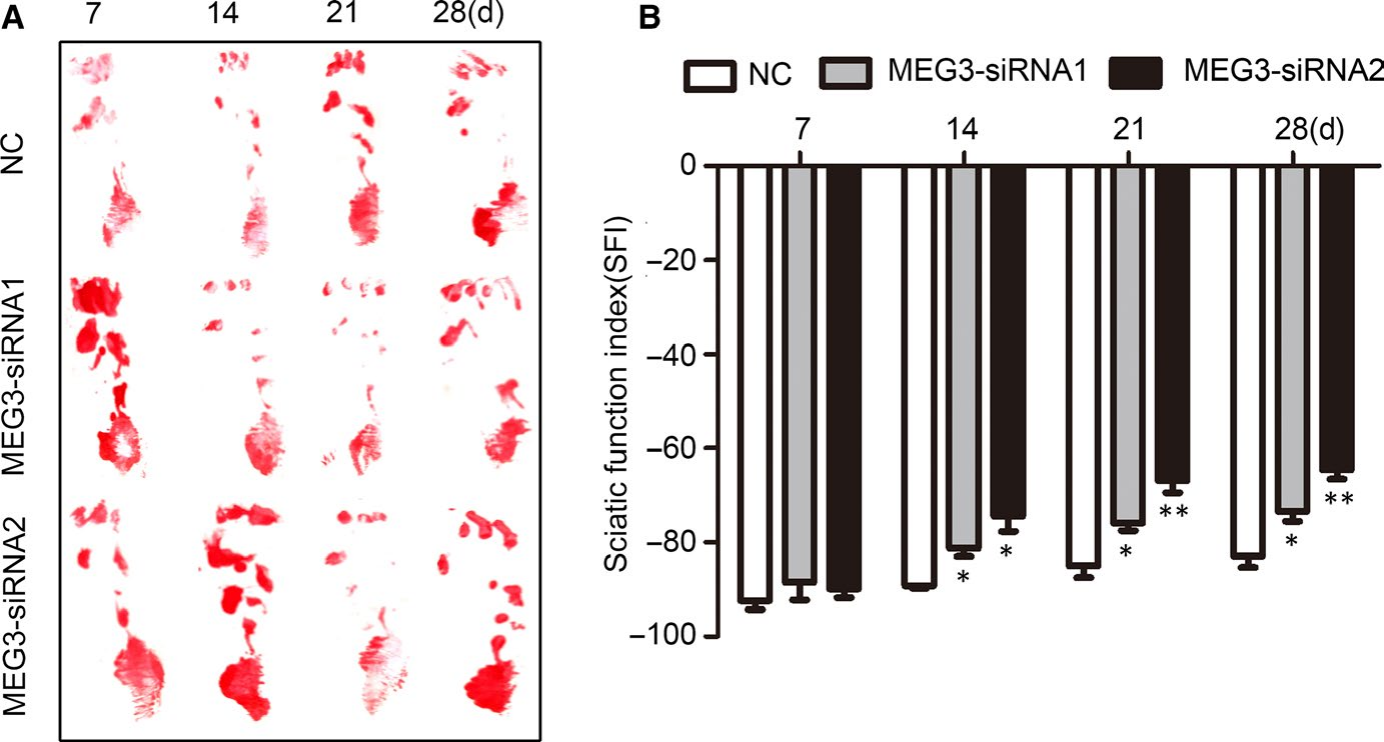
MEG3 affects the functional recovery after sciatic nerve injury. A, Representative pawprint from rats after sciatic nerve injury following MEG3 siRNA and control injection by walking track analysis at 7, 14, 21 and 28 d after surgery. B, Quantification of the SFI value at different time points from rats with sciatic nerve injury, n = 6. Values are presented as mean ± SEM. *P < 0.05, **P < 0.01

**FIGURE 7:**
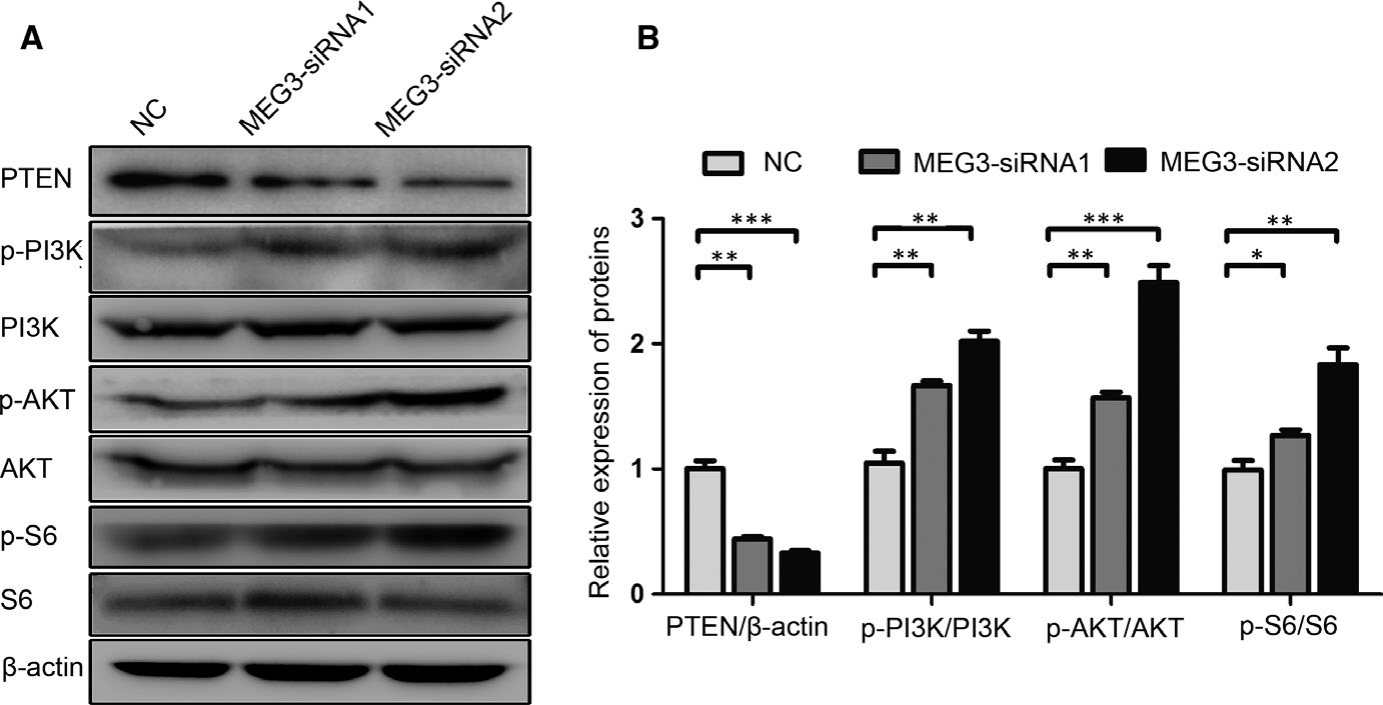
MEG3 regulates the PTEN/PI3K/AKT signalling in rats after sciatic nerve injury. A, Representative blot of PTEN, p‐PI3K, PI3K, p‐AKT, AKT, p‐S6 and S6 expression levels 7 days after sciatic nerve injury. B, Representative relative expression levels of the above proteins, n = 6. Values are obtained from three independent experiments and presented as mean ± SEM. *P < 0.05, **P < 0.01, ***P < 0.001

MEG3 regulates SC proliferation and migration and facilitates nerve regeneration. We further investigated whether its knockdown affects the functional recovery in rats after sciatic nerve transection. Walking track analysis and SFI were used to evaluate the functional recovery. As shown in Figure 6, the typical working tracks indicate an apparent improvement in MEG3 siRNA‐injected rats at 14 days after surgery. Similarly, the group injected with MEG3 siRNAs has significantly improved the SFI at 14 days after surgery compared with that in the NC control group (Figure 6B). The group injected with MEG3 siRNA has down‐regulated PTEN expression levels and up‐regulated p‐PI3K, p‐AKT and p‐S6 expression levels relative to those in the NC control group (Figure 7). These results are consistent with the finding that MEG3 regulates the proliferation and migration of SCs in vitro (Figure 4). Moreover, these results indicate that the knockdown of lncRNA MEG3 facilitates the functional recovery of the severed sciatic nerve by regulating the PTEN/PI3K/AKT pathway.

## 4 DISCUSSION

The proliferation and migration of SCs play a key role in peripheral nerve injury, provide trophic support to axons, and contribute to the development and regeneration of injured nerves.^20^ Inhibiting SC proliferation and migration by using local mitomycin can substantially decrease the axonal regeneration in rat sciatic nerve injury. Thus, exploring the regulatory mechanism of SCs is important for peripheral nerve injury therapy. lncRNAs are involved in nerve injury and regeneration.^5^, ^7^ Yao and colleagues reported that lncRNA TNXA‐PS1 modulates SCs by sponging miR‐24‐3p/miR‐152‐3p, thereby affecting the expression of dual‐specificity phosphatase1 and nerve regeneration in rat sciatic nerve injury.^14^ However, some roles of lncRNA in peripheral nerve injury are still unknown. In the present study, lncRNA MEG3 can affect nerve regeneration in a sciatic nerve‐transected rat by regulating the proliferation and migration of SCs in vitro and in vivo.

As a tumour suppressor gene, lncRNA MEG3 is the focus of tumour investigations and can inhibit the proliferation and metastasis of various tumour cells, including breast, lung, glioma, cervical, gastric and liver cancer cells.^21^, ^22^ This gene participates in neuropathological changes and nerve damage. Yi and colleagues reported that MEG3 expression is down‐regulated in the hippocampus tissues of rats with Alzheimer's disease (AD), and its up‐regulation can alleviate neuronal damage and improve the functional recovery via the PI3K/AKT signalling pathway.^23^ MEG3 expression is increased in patients with subarachnoid haemorrhage relative to that in healthy controls and participates in the SAH‐induced neuronal cell injury.^24^ However, the role of MEG3 in peripheral nerve damage and regeneration has not been reported.

In this study, the expression of lncRNA MEG3 is up‐regulated at 4 days and reached its peak 7 days after sciatic nerve injury. Moreover, the lncRNA MEG3 is expressed in SCs. Sciatic nerve injury can elicit transcriptional alteration.^25^ The time point of MEG3 up‐regulation shows a similar pattern to that of the expression of immune response genes, which are up‐regulated between 3 and 7 days following nerve crush injury and have peaked 7 days post‐crush.^26^ Results suggest that the lncRNA MEG3 participates in nerve injury and regeneration by regulating the proliferation and migration of SCs. These cells supplement the defective tissues through proliferation and migration and favour the axonal outgrowth and nerve regeneration following peripheral nerve injury.^27^ The primary SCs were cultured, and the localization of MEG3 in the SC cytoplasm was verified (Figure 2). Silencing lncRNA MEG3 improves the SC proliferation and migration via the PTEN/PI3K/AKT pathway (Figures 3 and 4). Notably, the peripheral nerve injury is often accompanied by ischemia and inflammation, resulting in the accumulation of reactive oxygen species (ROS).^28^ Aggregated ROS and inflammation have been reported to cause MEG3 up‐regulation in rats with cirrhotic neuropathy^29^ and UVB‐induced murine skin lesions.^15^ The knockdown of MEG3 improves the SC proliferation and migration and nerve function recovery in rats. This result is consistent with the observations that down‐regulated MEG3 expression protects against sciatic nerve injury in cirrhotic rat^29^ and UVB‐induced skin lesions in mice.^15^ Thus, the increase of lncRNA‐MEG3 at 7 days after sciatic nerve injury may be due to ROS and inflammatory factors produced after injury, but the mechanism needs further analysis.

After peripheral nerve injury, neurons activate regenerative or cell death signalling pathways, which may influence the injured neurons to regenerate or die.^30^ PTEN plays an important role in central axon growth as a negative regulator of the PI3K pathway,^31^ and its inhibition by using a specific PTEN phosphatase inhibitor or PTEN siRNA at the injury site facilitates peripheral nerve regeneration by accelerating axon outgrowth.^32^ Furthermore, the activation of the PI3K/Akt pathway promotes axon growth.^33^ The PI3K pathway activation in neurons by the exogenous growth factors or knockout of PTEN promotes neurite growth and elongation.^34^, ^35^ Consistent with the above report, MEG3 knockdown has down‐regulated the expression of PTEN, up‐regulated those of p‐PI3K, p‐AKT and p‐S6, and promoted SC proliferation and migration, axon outgrowth and the functional recovery of the injured sciatic nerves in rats (Figure 6). These results are also consistent with the role of MEG3 in cancer and AD. The overexpression of MEG3 inhibits endometrial cancer cell proliferation, invasion and metastasis via the PI3K pathway.^36^ Increased MEG3 expression inactivates the PI3/Akt pathway in the hippocampus tissues of rats with AD.^23^ lncRNAs also exert their regulatory role by sponging miRNA. lncRNA MEG3 sponges miR‐494 to inhibit haemangioma tumorigenesis by regulating the PTEN/PI3K/AKT pathway.^37^ The competitive binding of MEG3 and miR‐1297 to the 3′‐UTR of PTEN regulates the progression of testicular germ cell tumours via the PTEN/PI3K/AKT pathway.^38^ Thus, lncRNA MEG3 is inferred to participate in nerve repair and regeneration by regulating the proliferation and migration of SCs via the PTEN/PI3K/AKT signalling. However, the role of lncRNA MEG3 in neural injury needs to be further investigated to actively explore lncRNA‐ or ncRNA‐based therapies for peripheral nerve regeneration.

In summary, this study has revealed that lncRNA MEG3 regulates SC proliferation and migration in vitro and in vivo and participates in the nerve regeneration and functional recovery in sciatic nerve‐transected rats. Our findings suggest that MEG3 or other lncRNAs may be a promising therapeutic target for peripheral nerve injury.

## CONFLICT OF INTEREST

The authors declare that they have no competing interests. The funding agencies played no role in the design or implementation of the study, analysis or interpretation of the data, or the preparation and submission of the manuscript.

## AUTHOR CONTRIBUTIONS

YBM, LYD and XFW conceived and designed the experiments. YBM, DWZ, WZZ, HYZ, LYD and ZY performed the experiments. YBM, LYD, DQF, YPZ YZ and TW analysed the data**.** CMM and XFW contributed reagents/materials/analysis tools. YBM and XFW wrote the paper. All authors read and approved the final manuscript.

## Data Availability

The data that support the findings of this study are openly available in [repository name e.g ‘figshare’] at http://doi.org/[doi], reference number [reference number].
